# Dispersion-compensated Rowland spectrometer: implications for uranium VB-RIXS

**DOI:** 10.1107/S1600577525010318

**Published:** 2026-01-01

**Authors:** Martin Sundermann, Manuel Harder, Ayman H. Said, Bernhard Keimer, Hlynur Gretarsson

**Affiliations:** a Deutsches Elektronen-Synchrotron DESY Notkestr. 85 22607Hamburg Germany; b Max-Planck-Institut für Festkörperforschung Heisenbergstr. 1 70569Stuttgart Germany; c European XFEL Holzkoppel 4 22869Schenefeld Germany; d Max Planck Institute for Chemical Physics of Solids Nöthnitzer Straße 40 01187Dresden Germany; e Advanced Photon Source Argonne National Laboratory Lemont IL60439 USA; University of Essex, United Kingdom

**Keywords:** IRIXS beamline, PETRA III, DESY, VB-RIXS, RIXS, Rowland spectrometer

## Abstract

The total energy resolution (Δ*E*_tot_) of a valence-band resonant inelastic X-ray scattering (VB-RIXS) instrument serves as an important point of reference in an otherwise complex field. Since VB-RIXS is a flux-limited technique, a pragmatic approach to reducing Δ*E*_tot_ is often required—the specifications of a spectrometer should be matched with a comparable incident bandwidth (Δ*E*_i_) and the source size contribution (focal point) should be negligible. Although it advocates for a good efficiency, this approach is in many places already limited by count-rates. Here we follow a recent trend emerging in soft X-ray VB-RIXS and look at the performance of our tender X-ray Rowland spectrometer (Gretarsson *et al.*, 2020) when being exposed to a source with *a large linear dispersion* (higher flux). Detailed ray tracing work, performed at the U *M*_5_-edge (3551 eV), finds that the intrinsic resolution of the Rowland spectrometer (Δ*E*_a_) can be obtained if the linear dispersion of the source matches the spectrometer’s, but opposite in sign—here Δ*E*_i_ does not matter. This finding is supported by experimental data where Δ*E*_tot_ = 48 meV (Δ*E*_a_ = 44 meV) was recently achieved. Furthermore, we demonstrate that the dispersion rate can be tuned, ensuring the method’s applicability to other atomic edges.

## Abstract 2

A valence-band resonant inelastic X-ray scattering instrument, operating in the tender X-ray range, uses an incoming beam with a linear energy dispersion to reach the intrinsic resolution of the Rowland spectrometer. Such a scheme is insensitive to the incident bandwidth and therefore preserves count-rates despite the higher resolution.

## 1. Introduction

In the field of resonant inelastic X-ray scattering (RIXS) (Ament *et al.*, 2011 ▸), improvements in energy resolution (Δ*E*_tot_) have been impressive over the years. Both the benchmark *L*_3_-edges, Cu and Ir, have moved from ∼140 meV (Braicovich *et al.*, 2010 ▸; Kim *et al.*, 2012 ▸) to ∼30 meV (Rosa *et al.*, 2024 ▸; Kim *et al.*, 2014 ▸) thanks to the systematic developments in both monochromators and spectrometers. This has opened up new fields of science, including electron–phonon coupling in cuprates (Braicovich *et al.*, 2020 ▸) and spin-liquids in iridates (de la Torre *et al.*, 2023 ▸). Even research areas outside the traditional realm of strongly correlated electron systems have taken notice, with RIXS through the ligand absorption edges proving particularly successful (Marie *et al.*, 2024 ▸). Since these experiments detect low-energy excitations within valence bands (*E*_loss_ < 1 eV) they can be referred to as valence-band RIXS (VB-RIXS). This helps us set it apart from the related core-to-core RIXS (*E*_loss_ > 10 eV), a technique widely used to measure high-resolution X-ray absorption spectra (HERFD-XANES) (Bauer, 2014 ▸) and typically requires much less resolving power. For VB-RIXS, current optical approaches are nearing their count-rate limits, making it challenging to maintain these impressive developments. It is therefore timely to explore new optical approaches.

Overcoming the count-rate limitation has been a recurring theme in inelastic X-ray scattering for decades. First suggested by Schülke (1986 ▸), an incident bandwidth larger than Δ*E*_tot_ may be permitted as long as a dispersion compensation takes place. However, it is only recently that such an approach has been seriously considered for hard and soft X-ray inelastic techniques. Here the use of broadband X-rays (higher flux) enables ultra-high-energy resolution (Fung *et al.*, 2004 ▸; Lai *et al.*, 2014 ▸; Shvyd’ko, 2016 ▸; Shvyd’ko, 2017 ▸; Sánchez del Río & Shvyd’ko, 2019 ▸; Strocov, 2010 ▸; Zhou *et al.*, 2020 ▸; Singh *et al.*, 2021 ▸; Miyawaki *et al.*, 2022 ▸; Miyawaki *et al.*, 2025 ▸)—seemingly an oxymoron. In short, by spatially encoding the incident energy bandwidth (Δ*E*_i_) onto the sample (a rainbow strip), it is possible to design a spectrometer that can either image (Strocov, 2010 ▸) or compensate (Fung *et al.*, 2004 ▸; Shvyd’ko, 2016 ▸) for such an exotic source, effectively eliminating Δ*E*_i_ contributions to the total energy resolution of the instrument. Although this approach clearly deviates from current designs, which focus on achieving the smallest spot size with the narrowest bandwidth, such a beam can still be created using existing beamline components. For instance, both Singh *et al.* (2021 ▸) and Miyawaki *et al.* (2022 ▸) place the sample at the exit of a plane grating monochromator (soft X-rays), where a naturally occurring ‘rainbow’ source appears, while Chumakov *et al.* (2019 ▸) used asymmetrically cut crystals (hard X-rays), in combination with focusing optics, to achieve similar effects in the field of nuclear inelastic scattering. Furthermore, by imaging the elongated source, it is possible to retrieve the spatial resolution (Schunck *et al.*, 2021 ▸).

When it comes to spectrometers, the situation becomes more complex. To take advantage of the vertical ‘rainbow’ source, Singh *et al.* (2021 ▸) use an active grating (bendable) to compensate for the energy dispersion on the sample. In contrast, Miyawaki *et al.* (2022 ▸) rotate the spectrometer’s scattering plane from vertical to horizontal, enabling the vertical source to instead be imaged onto the detector while simultaneously dispersing the light horizontally. Although more compact, the proposed hard X-ray spectrometers require post-sample collimation, dispersion optics and focusing elements (Shvyd’ko, 2016 ▸; Shvyd’ko, 2017 ▸; Sánchez del Río & Shvyd’ko, 2019 ▸), a design that is radically different from the widely used Rowland spectrometer. Experimentally, such an approach is not as advanced as the so-called 2D-RIXS (Strocov, 2010 ▸; Zhou *et al.*, 2020 ▸) described above, partially because of the stringent specifications associated with hard X-rays (*e.g.* the very narrow Darwin widths). Given these two cases, exploring a beam with a linear energy dispersion would also be valuable for tender X-ray VB-RIXS.

**Figure 1:**
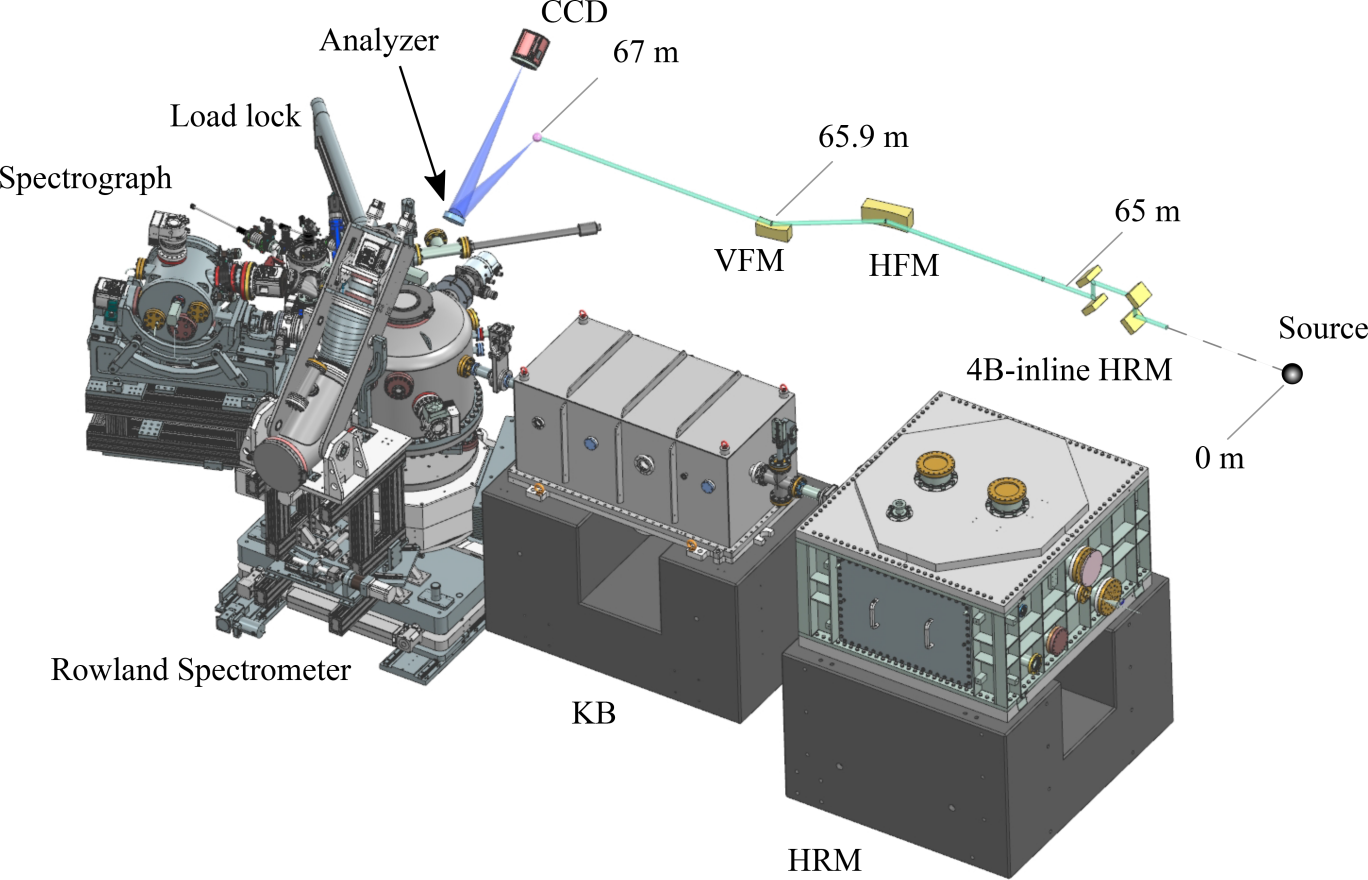
Drawing of the IRIXS instrument showing the position of some of its components from the source. These include a high-resolution monochromator (HRM), a Kirkpatrick–Baez (KB) mirror, a sample chamber and a Rowland spectrometer. In the adjacent layout the beam can be seen propagating from right to left, going through the multiple elements before hitting the sample and being subsequently analyzed by the spectrometer. The drawing also includes our spectrograph spectrometer as well as a load lock.

**Figure 2:**
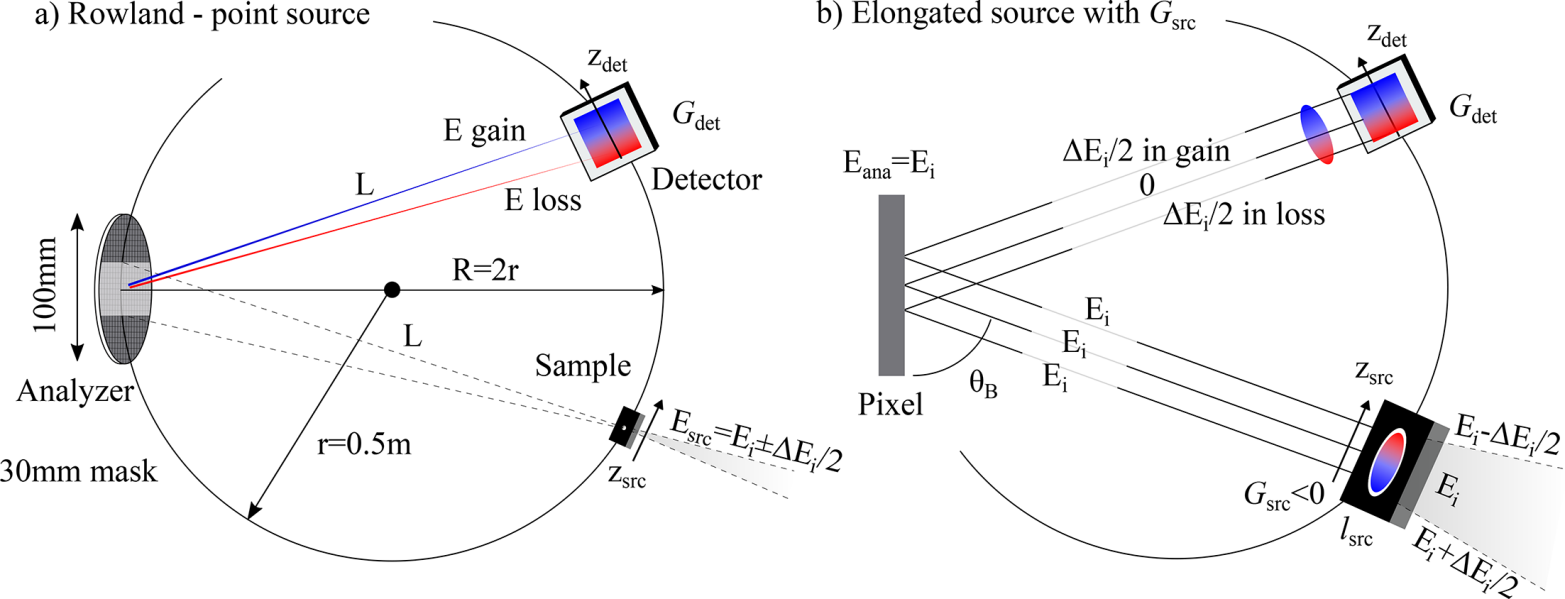
(*a*) A schematic of our Rowland spectrometer, showing sample, analyzer and detector on a common circle. *L* is the sample–analyzer and analyzer–detector distance. The analyzer is partially masked in the vertical direction (dark gray area) to limit the Johann error. The incident beam (source) has an energy range of *E*_src_ = *E*_i_ ± Δ*E*_i_/2. The linear dispersion on the detector [*G*_det_, see equation (1)] is depicted as blue (gain) and red (loss) colors. The local *z*-axis for both sample and detector is also shown. (*b*) Limited view of the now elongated source with a linear dispersion of *G*_src_ < 0 (higher *z*_src_ has a lower energy). A single analyzer pixel is used and all photons from the sample are parallel. Since the pixel only diffracts photons with an energy of *E*_i_ the single pixel effectively reverses the linear dispersion of the source. We go from Δ*E*_i_/2 in energy gain to Δ*E*_i_/2 in energy loss from the top to the bottom. By selecting *G*_src_ = −*G*_det_ a full linear dispersion compensation can occur (blue goes to blue) and intrinsic Rowland resolution is achieved.

The intermediate X-ray energy RIXS (IRIXS) instrument at beamline P01 PETRA-III DESY (Gretarsson *et al.*, 2020 ▸) operates in the tender X-ray range (2.4–4.0 keV), covering the energies of many 4*d* transition metal *L*_2,3_-edges as well as uranium *M*_4,5_-edges (see Fig. 1 ▸, with a drawing and a layout of the instrument). One of its prime objectives is to offer high energy resolution VB-RIXS (Δ*E*_tot_ < 100 meV), providing an important option to the more common core-to-core RIXS (Δ*E*_tot_ ≃ 0.5–1 eV) available in the tender range at multiple beamlines (Rovezzi *et al.*, 2020 ▸; Scheinost *et al.*, 2021 ▸; Vitova *et al.*, 2017 ▸; Tobin *et al.*, 2022 ▸). In terms of main optics, IRIXS has a high-resolution monochromator (HRM), based on a series of silicon crystals (see Fig. 4 for details), a Kirkpatrick–Baez (KB) mirror (its vertical mirror has a focal distance of *l*_KB_ = 1.1 m), and a Rowland-type spectrometer [*R* = 1 m, see Fig. 2 ▸(*a*) for more details], equipped with a spherically diced and bent analyzer (either α-SiO_2_ or LiNbO_3_). Although IRIXS is a state-of-the-art instrument, further improving its energy resolution—just as with existing VB-RIXS beamlines—remains a challenge. To this extent, we have tested a spectrograph spectrometer that gives 35 meV at the Ru *L*_3_-edge (2840 eV) (Bertinshaw *et al.*, 2021 ▸) (included in the drawing of Fig. 1 ▸). However, this post-sample collimation solution is limited in working energy (*e.g.* Montel mirror gives only 2–5% energy bandwidth) and delivers low count-rates due to the large number of optical elements. As with other highly specialized spectrometers (Kim *et al.*, 2016 ▸; Kim *et al.*, 2018 ▸; Kim *et al.*, 2020 ▸), the spectrograph is designed for a narrow, yet scientifically important, set of applications. On the other hand, the Rowland approach (Gretarsson *et al.*, 2020 ▸) covers multiple edges and delivers sufficient flux to the detector but has restricted upside in resolution due to the scarcity of crystal analyzer. For example, at the Ru *L*_3_-edge, an α-SiO_2_(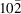) analyzer is used which gives intrinsic spectrometer/analyzer resolution of Δ*E*_a_ = 64 meV—twice as large as that of the spectrograph. In contrast, at the U *M*_5_-edge (3551 eV), an α-SiO_2_(003) analyzer achieves a more impressive Δ*E*_a_ = 44 meV. Given the experimental constraints, eliminating the incident bandwidth contribution to Δ*E*_tot_, by working with a ‘rainbow’ source, could further enhance IRIXS performance without compromising good count-rates.

In this article, we investigate the impact of the so-called ‘rainbow’ source on the performance of our Rowland RIXS spectrometer (Gretarsson *et al.*, 2020 ▸). We begin by presenting a simplified general case to introduce the relevant parameters and then demonstrate that such a source can positively affect energy resolution, provided it has the correct linear dispersion. This is followed by extensive ray-tracing simulations using *XRT* (Klementiev & Chernikov, 2014 ▸), where we show that Δ*E*_tot_ = Δ*E*_a_ can be achieved at the U *M*_5_-edge irrespective of the incident bandwidth. As a consequence of the finite linear dispersion rate, the best possible resolution is therefore not achieved with the smallest focal spot, but with a large, elongated beam. Lastly, we present the experimental values, where we achieve a resolution of Δ*E*_tot_ = 48 meV for uranium—just 5 meV higher than the intrinsic resolution of the analyzer. Moreover, we explain how the ‘rainbow’ can be tuned, by varying the asymmetric parameters of the high-resolution monochromator, in order to apply this solution to other atomic edges within the tender energy range.

## 2. A hidden dispersion compensation

A Rowland spectrometer places a sample, an analyzer and a detector on a common circle [see Fig. 2 ▸(*a*)]. In our case, the radius is *r* = 0.5 m and a spherically bent (*R* = 2*r*) and diced analyzer is used (pixel length is *l*_ana_ = 1.5 mm). The analyzer collects scattered photons from the sample and diffracts them, in the vertical plane, onto a position sensitive 2D detector with a pixel width of *l*_det_ = 13.5 µm. For a point source, the linear dispersion rate (µm meV^−1^) of the detector (*G*_det_) is calculated with Bragg’s law and is equal to 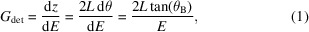
where *L* = 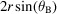 is the sample–analyzer (analyzer–detector) distance and θ_B_ is the Bragg angle of the analyzer for an energy of *E*. When *E* matches the energy range of the source (*E*_i_ ± Δ*E*_i_/2) the spectrometer is looking at the elastic line. However, due to the finite size of *l*_ana_ the spectrometer sees a large spectral window of 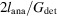. We note that, thanks to the position sensitive detector, this large spectral window (in our case ∼1 eV) does not contribute to energy broadening, as shown by Shvyd’ko *et al.* (2012 ▸). With *G*_det_ > 0, the formula demonstrates that at a higher *z*_det_ value a higher energy (blue color) is found. The energy resolution of this type of spectrometer, with both a well focused source and a small *l*_det_, is in the end limited by the bandwidth of the spectrometer/analyzer (Δ*E*_a_) and the bandwidth of the incident X-rays (Δ*E*_i_). In the remainder of this paper, we will show that surpassing this so-called hard limit is both feasible and practical in the tender X-ray range.

Using a vertical ‘rainbow’, instead of a point source, has unexpected implications. To simplify the task only a single pixel at the center of the analyzer is considered [Fig. 2 ▸(*b*)] with all the photons from the sample being parallel. For the source, a negative dispersion rate was chosen (*G*_src_ < 0) so that at the highest *z*_src_ value we get an energy of *E*_i_ − Δ*E*_i_/2 (red color). In terms of the beam scattered from the sample it is sufficient to draw three lines coming out: top, center and bottom. The single analyzer pixel only reflects photons with an energy of *E*_i_ which results in an interesting energy loss/gain profile of the diffracted beam hitting the detector—we now have the reverse source profile. To elaborate, the bottom of the source has an energy of *E*_i_ + Δ*E*_i_/2. The only way that a photon gets reflected is if it can give Δ*E*_i_/2 to the sample, bringing the energy down to *E*_i_. Therefore, this photon, with an absolute energy of *E*_i_, effectively represents an energy loss of Δ*E*_i_/2 instead of 0 (elastic line). Based on this we can say that, if the linear dispersion rate of the reflected beam matches the detector’s, we ensure that all photons go into their correct energy loss/gain ‘boxes’. Subsequently we eliminate both Δ*E*_i_ as well as the source size contributions. In other words, the following simple equation must be satisfied,
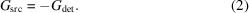
This simple exercise also holds for any other energies (*E* ≠ *E*_i_) provided that they can be Bragg reflected. By looking at some numbers we can demonstrate that equation (2) provides realistic values. For instance, we get *G*_det_ ≃ 2 µm meV^−1^ for both Ru *L*_3_-edge (2840 eV) and U *M*_5_-edge (3551 eV), using α-SiO_2_(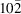) and α-SiO_2_(003), respectively. With such a number the height of the source would become *l*_src_ = |Δ*E*_i_ × *G*_src_| ≃ 50 meV × 2 µm meV^−1^ = 100 µm, if we assume Δ*E*_i_ = 50 meV. We note that, while larger than the usual vertical beam size of ∼10 µm in RIXS (Moretti Sala *et al.*, 2018 ▸), such a beam is still small enough for most crystals.

## 3. Simulations

**Figure 3:**
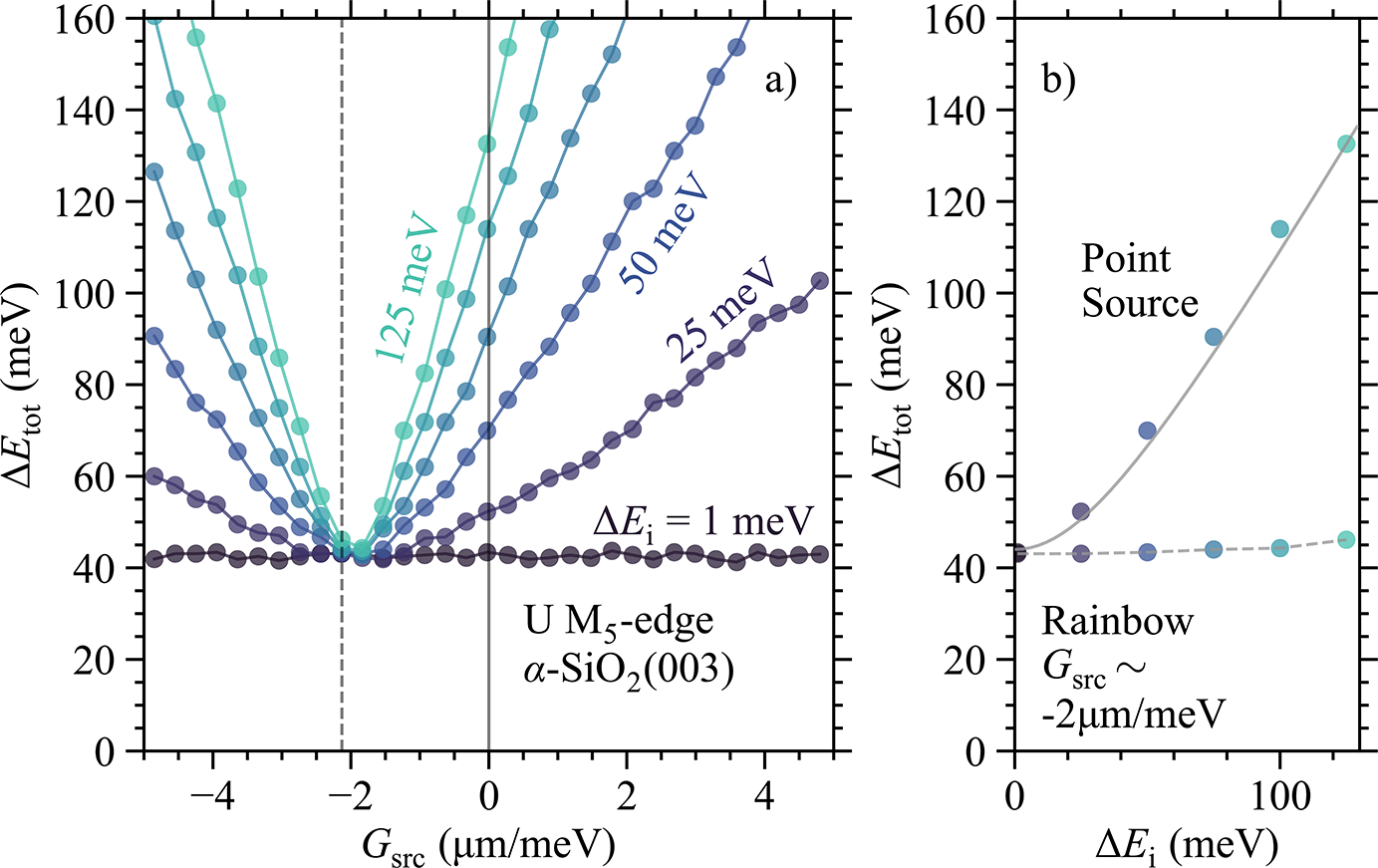
(*a*) Numerical simulations showing the total energy resolution of our RIXS instrument as a function of linear dispersion of the source and incident bandwidth. We use the Rowland spectrometer setup in Fig. 2 ▸(*a*) with an α-SiO_2_(003) analyzer and incident energy tuned to the U *M*_5_-edge (3551 eV). A clear minimum is observed away from the point-source limit (*G*_src_ = 0), largely in agreement with equation (2). (*b*) Vertical cuts through the minimum and the point-source in (*a*), dashed and solid line, respectively. This depicts how the total energy resolution depends on the incident bandwidth. The data for the point-source is overlaid with the expected behavior of Δ*E*_tot_ = 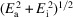 for a Gaussian error distribution. Here *E*_a_ = 44 meV.

To verify that equation (2) is valid for a realistic Rowland spectrometer setup (*e.g.* a finite Darwin width) we carried out extensive ray tracing work at the U *M*_5_-edge (3551 eV) using α-SiO_2_(003) as an analyzer. In Fig. 3 ▸(*a*) we show our findings, where Δ*E*_tot_ is plotted as a function of *G*_src_ for various Δ*E*_i_ values. Results for Δ*E*_i_ = 1 meV can be considered as the intrinsic resolution of the spectrometer/analyzer with Δ*E*_a_ = 44 meV, which takes into account both the intrinsic resolution of α-SiO_2_(003) (35 meV) as well as the finite size of the analyzer mask in Fig. 2 ▸(*a*). With a Bragg angle of θ_B_ ≃ 76° we are still some way from true backscattering geometry, which means the Johann error is present—though significantly minimized.

For larger values of Δ*E*_i_ there exists a clear minimum in Fig. 3 ▸(*a*) which, interestingly, is away from the well known point-source (*G*_src_ = 0) and is indeed located on the negative side. The minimum is close to a value of *G*_src_ ≃ −2 µm meV^−1^ (dashed vertical line) predicted from equation (2) and reaches the intrinsic resolution of the spectrometer (Δ*E*_tot_ ≃ Δ*E*_a_ ≃ 44 meV), regardless of Δ*E*_i_. This highlights the benefit of the hidden linear dispersion compensation.

In Fig. 3 ▸(*b*) we show two vertical cuts from Fig. 3 ▸(*a*) (dashed and solid lines). If one sticks to a point source, Δ*E*_i_ = 50 meV would be required to obtain good efficiency, which leads to Δ*E*_tot_ = 70 meV. By reducing the bandwidth to 25 meV, we move closer to the intrinsic resolution of the spectrometer. However, this comes at a significant cost to the flux, as the spectral reflectivity of a *higher*-resolution monochromator typically also decreases—compounding the loss already incurred from the narrower bandwidth. Using an actual example from the Ru *L*_3_-edge, going from Δ*E*_i_ = 60 meV to 30 meV reduced the flux by 1/6 (bandwidth is 1/2 and reflectivity is 1/3) (Bertinshaw *et al.*, 2021 ▸). Likewise, simulations for the U *M*_5_-edge show a reduction in flux by 1/8 (bandwidth is 1/2 and reflectivity is 1/4) when going from Δ*E*_i_ = 50 meV to 25 meV. This means that the new optical scheme can yield almost an order of magnitude higher count rates compared with the classical case. In Fig. 3 ▸(*b*) we also plot the expected behavior of Δ*E*_tot_ = 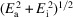 for a point source and whose errors have a Gaussian distribution (solid line). A relatively good agreement is found. In contrast, the ‘rainbow’ source can work with practically any incident bandwidth as long as the linear dispersion is correct.

## 4. Experiment

The ray tracing work established a hidden linear dispersion compensation inside the Rowland spectrometer. To take advantage of this feature we describe how the source can be tailored to fulfill equation (2) using a similar approach to Chumakov *et al.* (2019 ▸).

**Figure 4:**
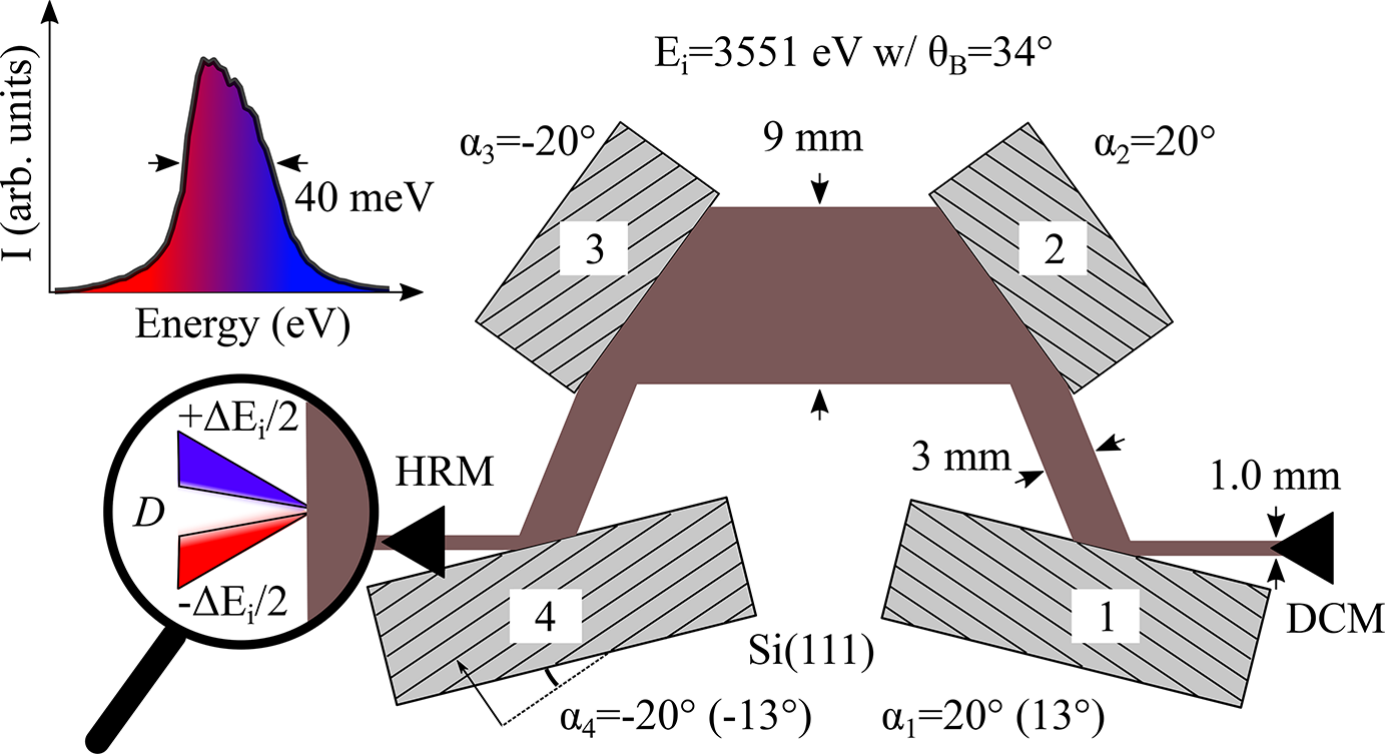
A schematic of the four-bounce inline HRM (setup 1) with angles mimicking the U *M*_5_-edge configuration. The HRM consist of four asymmetrically cut (α) Si(111) crystals with α_1_ = − α_4_ (α_2_ = −α_3_). Two different configurations are possible and we refer to them as HRM setup 1 (α_1, 2_ = 20°) and setup 2 (α_1_ = 13° and α_2_ = 20°), respectively. The multiple black solid lines on each crystal represent the orientation of the Si(111) lattice planes. The large dispersion rate (*D*_src_ = 3.5 rad meV^−1^ at U *M*_5_-edge for setup 1), produced by the HRM, is depicted inside the magnifying glass as blue/red beams pointing up/down. The inset shows the calculated energy profile at the U *M*_5_-edge for setup 1.

At IRIXS, the incident bandwidth can be monochromated using a four-bounce inline HRM equipped with asymmetrically cut Si(111) crystals (see Fig. 4 ▸) (Gretarsson *et al.*, 2020 ▸). Here the asymmetry angle (α) is defined as the angle between the Si(111) reflection plane and the surface and α_1_ = −α_4_ (α_2_ = − α_3_). As discussed by Huang *et al.* (2012 ▸), this type of a monochromator introduces an unwanted angular dispersion rate *D*_src_ = Δθ_i_/Δ*E*_i_ to the beam (Δθ_i_ is the angular divergence after the HRM) but here we take advantage of this. In Fig. 4 ▸ the magnifying glass demonstrates how this effect appears along the vertical HRM beam profile. Higher (lower) energies point upwards (downwards). The formula for the cummulative dispersion rate of a four-bounce inline HRM was derived by Shvyd’ko (2015 ▸) and can in our case be calculated using
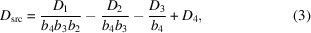
where the individual asymmetric parameters and dispersion rates are defined as *b*_*n*_ = 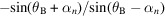 and *D*_*n*_ = 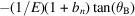 with *n* = 1, 2, 3 and 4. A linear dispersion can then be created by simply focusing the beam vertically, 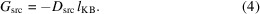
Here, the minus comes from the upward deflection and *l*_KB_ = 1.1 m is again the focal length of the vertical mirror. To fulfill equation (2) we therefore need *D*_src_ = 1.9 µrad meV^−1^ which can be achieved using α_1_ = 11° and α_2_ = 20°.

To put this to the test we have equipped our four-bounce inline HRM with a second configurations for α. In addition to having α_1,2_ = 20° (HRM setup 1) we also have α_1_ = 13° and α_2_ = 20° (HRM setup 2). While setup 1 was designed to give a bandwidth of 60 meV at the Ru *L*_3_-edge, setup 2 was designed to get closer to fulfilling equation (2) at the U *M*_5_-edge. Indeed, using equations (3) and (4), we get *G*_src_ = −4.3 and −2.5 µm meV^−1^ for these two setups, which gives us enough contrast to observe the dispersion compensation effect in Fig. 3 ▸(*a*).

**Figure 5:**
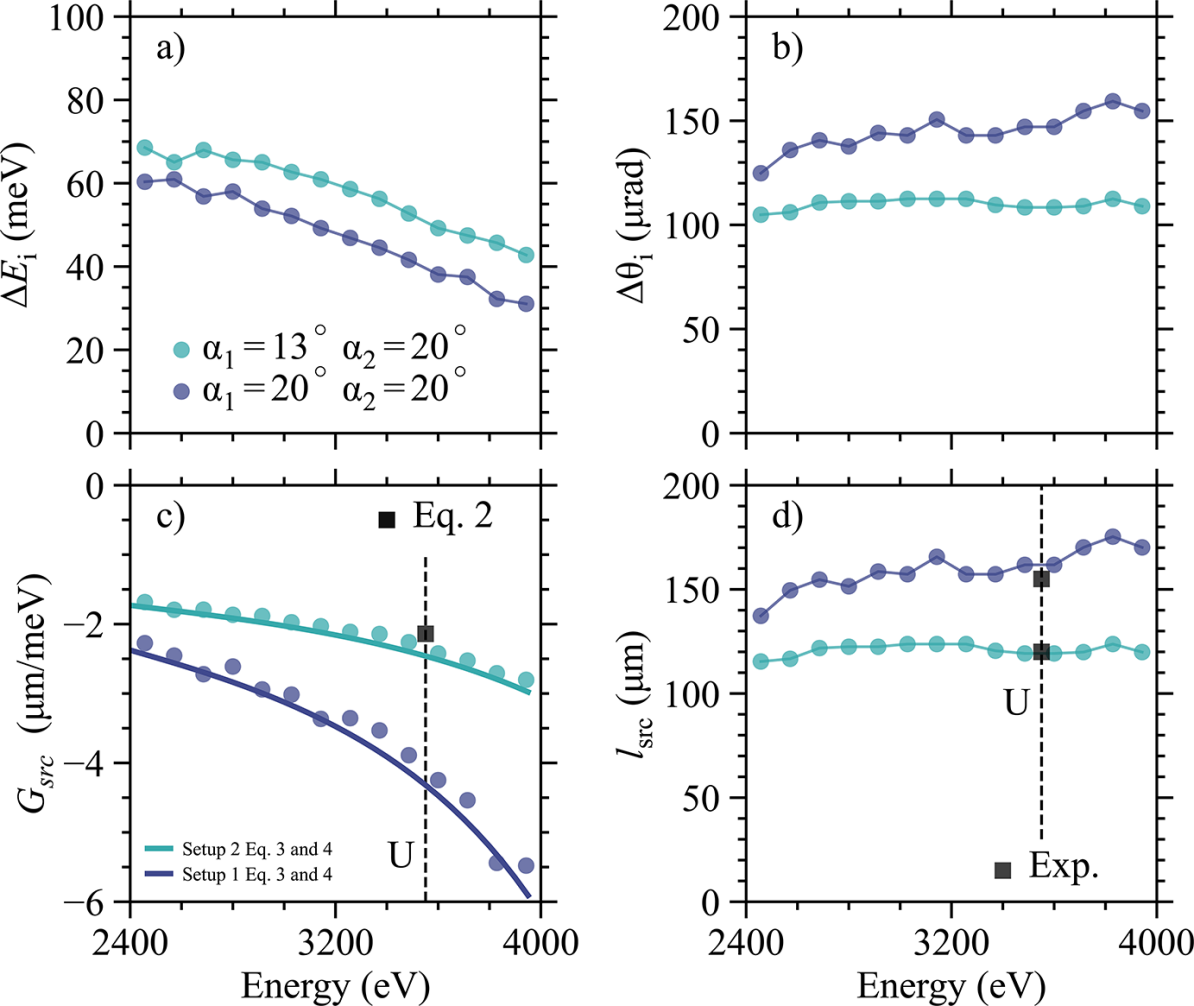
Ray tracing results showing the expected (*a*) incident bandwidth and (*b*) divergence from the 4B-inline HRM over the working energy range. Results are shown for two different HRM setups (see text). In (*c*) and (*d*) we plot the expected linear dispersion rate and source size when the HRM beam is focused. Thick solid lines in (*c*) are derived from equation (3). The square symbol in (*c*) represents the linear dispersion required from equation (2) while in (*d*) the experimental values for the vertical focus of the two different HRM setups.

In Fig. 5 ▸ we present ray-tracing results for these two setups over an energy range of 2.4–4.0 keV, plotting both the incident (*a*) bandwidth and (*b*) divergence. The inset in Fig. 4 ▸ shows the simulated energy profile at the U *M*_5_-edge with Δ*E*_i_ = 40 meV (setup 1), where blue is again used for higher energies. Due to the large absorption in the tender X-ray range the energy profile is fairly asymmetric. In Figs. 5 ▸(*c*) and 5(*d*) we plot the linear dispersion rate [using equation (4)] as well as the predicted vertical spot size over the same energy range using *l*_src_ = |Δ*E*_i_*G*_src_|. Since the angular divergence of the beam leaving the HRM is much larger than the its natural divergence the formula for *l*_src_ is justified. In Fig. 5 ▸(*c*) the values derived from equation (3) are plotted as thick solid lines for comparison, showing only slightly larger negative values compared with the ray tracing results.

Two things are worth highlighting from the numbers presented in Fig. 5 ▸. First, as expected, by reducing α_1_ from 20° to 13°, the bandwidth gets larger and the divergence decreases over the entire range. As a result, these changes give rise to a smaller *G*_src_ which improves the focus as well, going from ∼150 to ∼120 µm. Additionally, the larger bandwidth and better reflectivity (lower α_1_) leads to a higher photon flux—at the U *M*_5_-edge, setup 2 gives ∼1.5× the photons compared with setup 1. Second, following equation (2), we need *G*_src_ = −2.1 µm meV^−1^ for the U *M*_5_-edge, a number that is obviously closer to being reached with setup 2 [see the filled black square in Fig. 5 ▸(*c*)].

**Figure 6:**
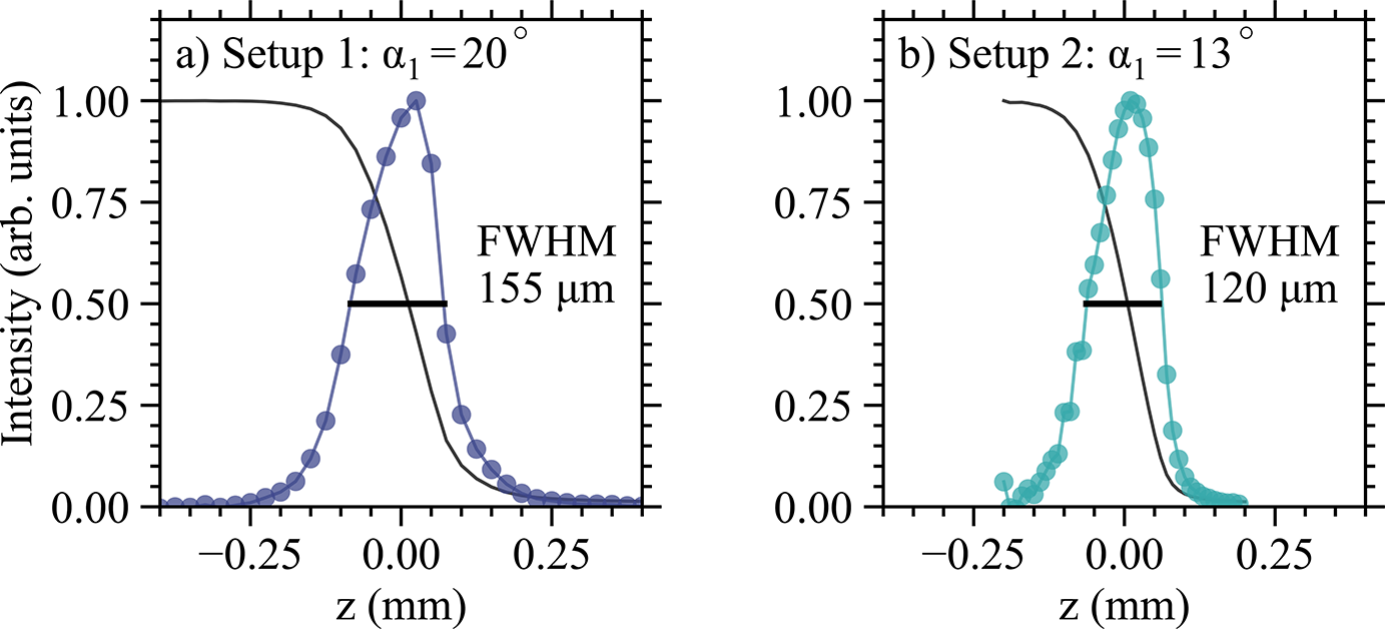
The vertical size of the focused beam on the sample. Data are taken at the U *M*_5_-edge using two different HRM setups. The solid line is from a blade scan while the circles are its first derivative. The thick horizontal lines represent the estimated full width at half-maximum (FWHM). Fitting was avoided due to a large asymmetry in the beam profile.

The easiest way to verify the results in Fig. 5 ▸ is to measure the actual focus for setup 1 and 2 at the U *M*_5_-edge. Results are plotted in Fig. 6 ▸ and show that the size of the beam for setup 1 is 155 µm while for setup 2 is 120 µm, values that are in good agreement with Fig. 5 ▸(*d*) (see solid black squares). Additionally, one notices a pronounced asymmetry of the beam profile for setup 1 which closely resembles the energy profile in the inset of Fig. 4 ▸. This indicates that higher energies are indeed found near the bottom of the focused beam (*G*_src_ < 0). In Appendix *A*[App appa], ray tracing work is provided for setup 1, leading to the same conclusion. Taken together, this observation strongly supports the existence of a vertically elongated ‘rainbow’ beam.

**Figure 7:**
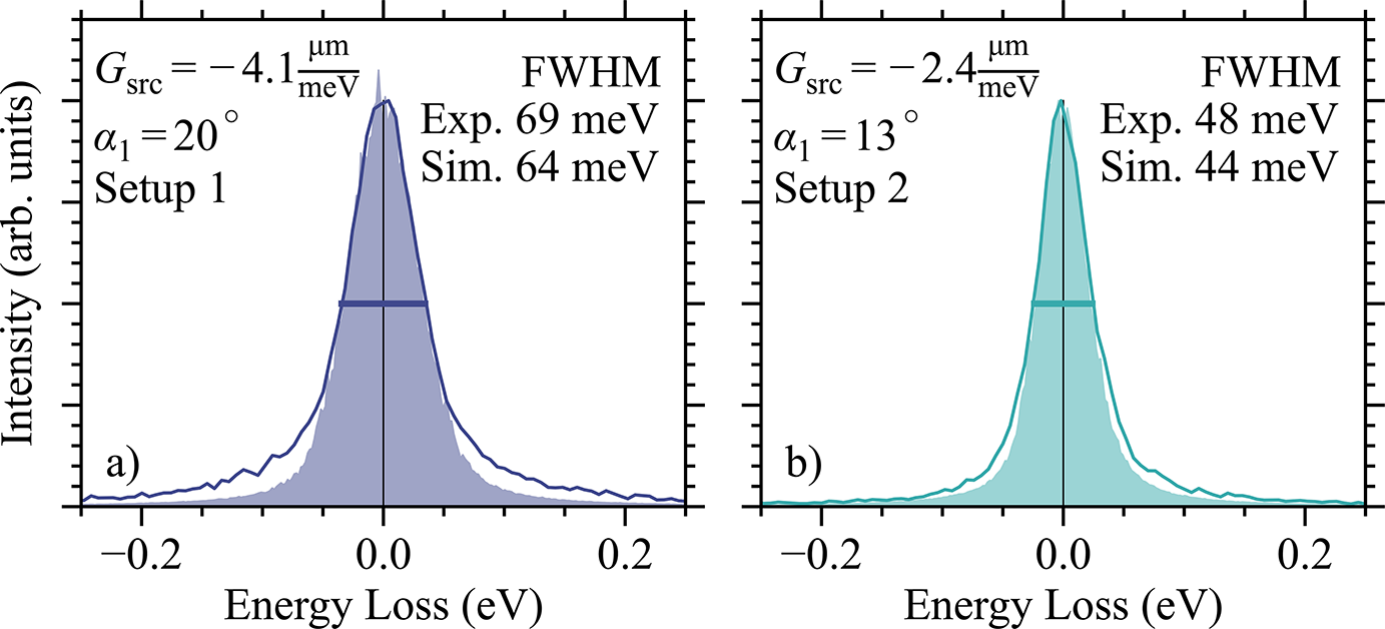
Experimentally measured elastic scattering (solid lines) in comparison with simulations (filled area). Two different HRM setups were used for the U *M*_5_-edge and an α-SiO_2_(003) as the analyzer. Data were collected by measuring photons scattered off a piece of carbon tape. Due to mechanical limitations the spectrometer’s 2θ was set at 90°, which unfortunately, minimizes elastic scattering.

**Figure 8:**
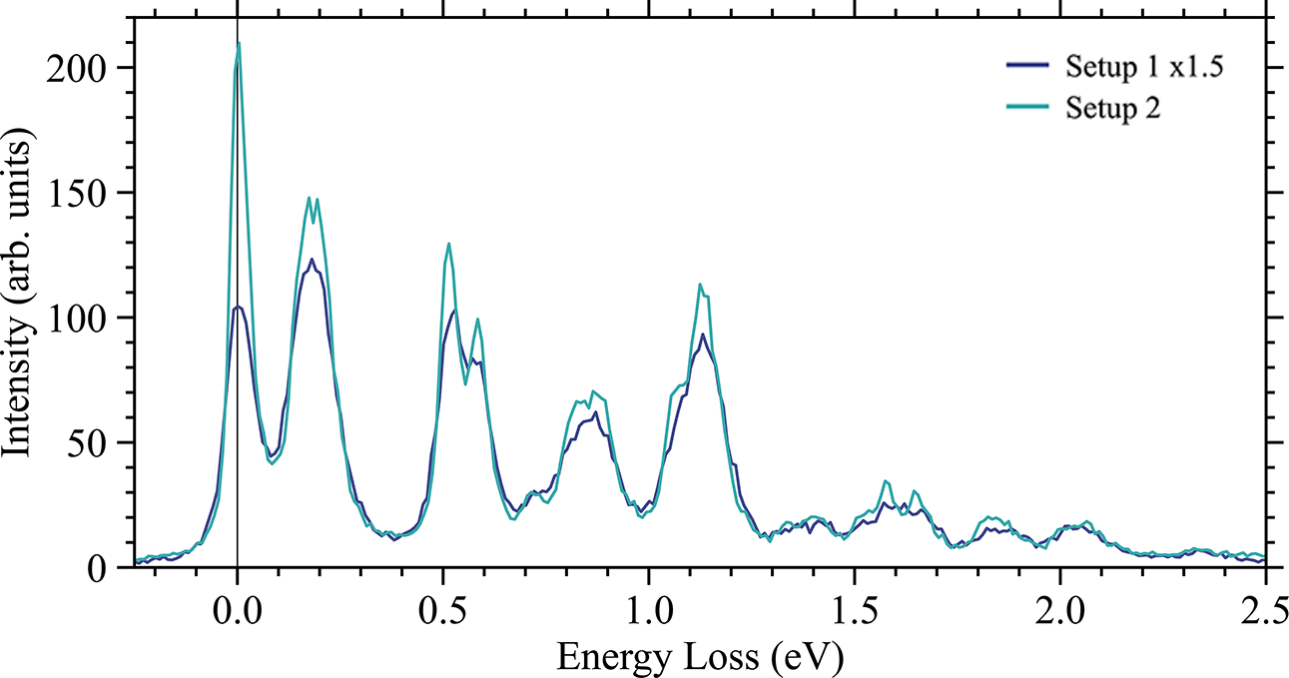
Measurements on a single crystal of UO_2_ using RIXS at the U *M*_5_-edge. Data were collected using two HRM setups. The spectrum of setup 1 has been scaled by 1.5 to account for the lower photons flux. A good overlap is observed for the background and the better resolution of setup 2 is evident in overall sharper peaks. Data for setup 2 were taken from Sundermann *et al.* (2025 ▸).

Having characterized the incident beam and shown that it fits our criteria we can look at the experimentally determined Δ*E*_tot_ values in Fig. 7 ▸. An un-etched spherically bent and diced α-SiO_2_(003) analyzer (θ_B_ ≃ 76°) was used for this test. For more details on the analyzer fabrication, see Ketenoglu *et al.* (2015 ▸). Data were collected with the spectrometer 2θ = 90° and a carbon tape was used as a scatterer. This geometry is unfavorable for elastic scattering due to the Thomson scattering factor [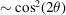] but could not be changed for mechanical reasons (the entire beamline, including the spectrometer, is in vacuum). Nevertheless, the quality allows us to compare our values with ray tracing results. For setup 1 we have *G*_src_ = −4.1 µm meV^−1^ while setup 2 gives −2.4 µm meV^−1^, with the latter number being much closer to the minimum in Fig. 3 ▸(*a*). In line with that statement, we see a big improvement in Δ*E*_tot_ when changing to setup 2, going from 69 meV to 48 meV, a number that is largely in agreement with what our simulations give (filled area). We note that large tails are presented in Fig. 7 ▸ which are less pronounced in the simulations; this likely stems from some imperfection of the analyzer that are more visible in this geometry (2θ = 90°). However, we stress that the improvement from (*a*) to (*b*) takes place despite the larger incident bandwidth in HRM setup 2 (50 meV versus 40 meV), which in itself is remarkable. Further improvement towards 35 meV could be made by reducing the analyzer’s mask size by half (30 mm to 15 mm in height) and changing α_1_ to be 11°. In Fig. 8 ▸ we demonstrate both the increased count rates as well as the improved resolution by measuring a single crystal of the semiconducting UO_2_ using U *M*_5_-edge VB-RIXS. Data for setup 1 have been scaled by 1.5 to account for the lower photon flux. Multiple inelastic features, associated with *ff*-excitations of uranium, can be observed up to 2.5 eV in energy loss (Sundermann *et al.*, 2025 ▸). For setup 2 they are not only more intense but also sharper due to the improved resolution. This small exercise therefore demonstrates the utility of our method.

## 5. Discussion

RIXS spectrometers are usually designed around monochromatic and focused beams, where the total resolution can be estimated based on 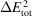 = 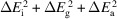. Here Δ*E*_g_ comes from geometrical contributions such as the source or the detector’s pixel size (Δ*E*_pixel_). However, our work shows that for the well known Rowland spectrometer there are hidden advantages in using broadband and elongated beams. This holds as long as the energy is spatially encoded with a linear dispersion rate that is opposite to the detector’s [see equation (2)]. In that scheme the estimated resolution can be reduced to Δ*E*_tot_ = Δ*E*_a_, given that Δ*E*_pixel_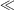 Δ*E*_a_. For a reference, we get Δ*E*_pixel_ ≃ 7 meV at the U *M*_5_-edge which validates this simplification.

For IRIXS this finding is particularly important since providing a monochromatic beam in the 2.4–4.0 keV range necessitate the use of an inline 4B-HRM. To elaborate, for the Ru *L*_3_-edge we have explored a *dispersionless* nested 4B-HRM (Bertinshaw *et al.*, 2021 ▸) which delivers an impressive Δ*E*_i_ = 30 meV. Having no energy dispersion (*D*_src_ = 0) means that a well focused beam (<20 µm) can be reached, which has implications for microcrystals (no spillover). Nevertheless, the nested 4B-HRM has reduced flux (1/6) and its working energy is by design very limited (<100 eV). This makes operation for other edges not practical. Asymmetrically cut Si(111) artificial channel cuts have also been tested but their bandwidth does not get close to our requirements. Moving to α-SiO_2_ is also not an option since quartz is unstable in direct beam (Gog *et al.*, 2018 ▸). Therefore, if we stay with conventional HRM designs, the inline HRM appears to be our only viable option. In this context, the ability to transform its well known drawback into an advantage becomes crucial.

In the tender X-ray range limited analyzers are available due to the scarcity of high quality single crystals with a large area and matching lattice constant, which can make some interesting systems very difficult to study. In Table 1 ▸ (see Appendix *B*[App appb]) a list of potential IRIXS edges is presented along with the most suitable analyzers (given our mechanical limitations). As one can see, the U *M*_5_-edge provides one of the best cases, which highlights another important point of our findings. Being able to reach the intrinsic spectrometer/analyzer resolution, without sacrificing the flux, makes our otherwise challenging situation somewhat easier to live with. It also has implications for edges that have even lower θ_B_, where reducing the analyzer’s mask further (less Johann) would have a big impact. At the U *M*_4_-edge, we have θ_B_ ≃ 67° which means going from a 30 mm to a 15 mm analyzer mask can make a big difference. This however cuts the count-rates in half but would be compensated by reducing the asymmetry angle of the HRM according to the value in Table 1 ▸. As a result, one could expect to reach the same resolution at both U *M*_5_- and *M*_4_-edges.

More broadly, this hidden dispersion compensation paves the way for exploring more exotic analyzers in the future, not only for tender but also hard X-rays (Kim *et al.*, 2024 ▸). Analyzers whose intrinsic resolution would otherwise demand severely reduced flux—due to the need for a significantly smaller Δ*E*_i_—could become viable options. Similarly, analyzers whose wafers are difficult to fabricate in large sizes would also become more attractive since the incident flux can be enhanced to make up for the lower solid angle. As for the scientific significance of our results, the high energy resolution reportered here for the U *M*_5_-edge VB-RIXS will provide the large core-to-core RIXS community with another tool to study valence states of uranium based compounds, address questions regarding covalancy, and even determine crystal-field splitting within multiplets, results that provide crucial input into theoretical models describing these facinating and technologically important 5*f* electron systems.

## 6. Summary

A scheme to reach the intrinsic resolution of a tender X-ray VB-RIXS spectrometer/analyzer (Δ*E*_a_) was described in detail. This requires the linear dispersion rate of the incident beam to match the detector’s in magnitude, but be opposite in sign. A test was done using the U *M*_5_-edge (3551 eV) where a record energy resolution of Δ*E*_tot_ = 48 meV was reached, which deviates by less than 5 meV from Δ*E*_a_. This method can be used to improve the energy resolution at other atomic edges in the tender range and, importantly, is independent of the incident bandwidth.

## Figures and Tables

**Figure 9:**
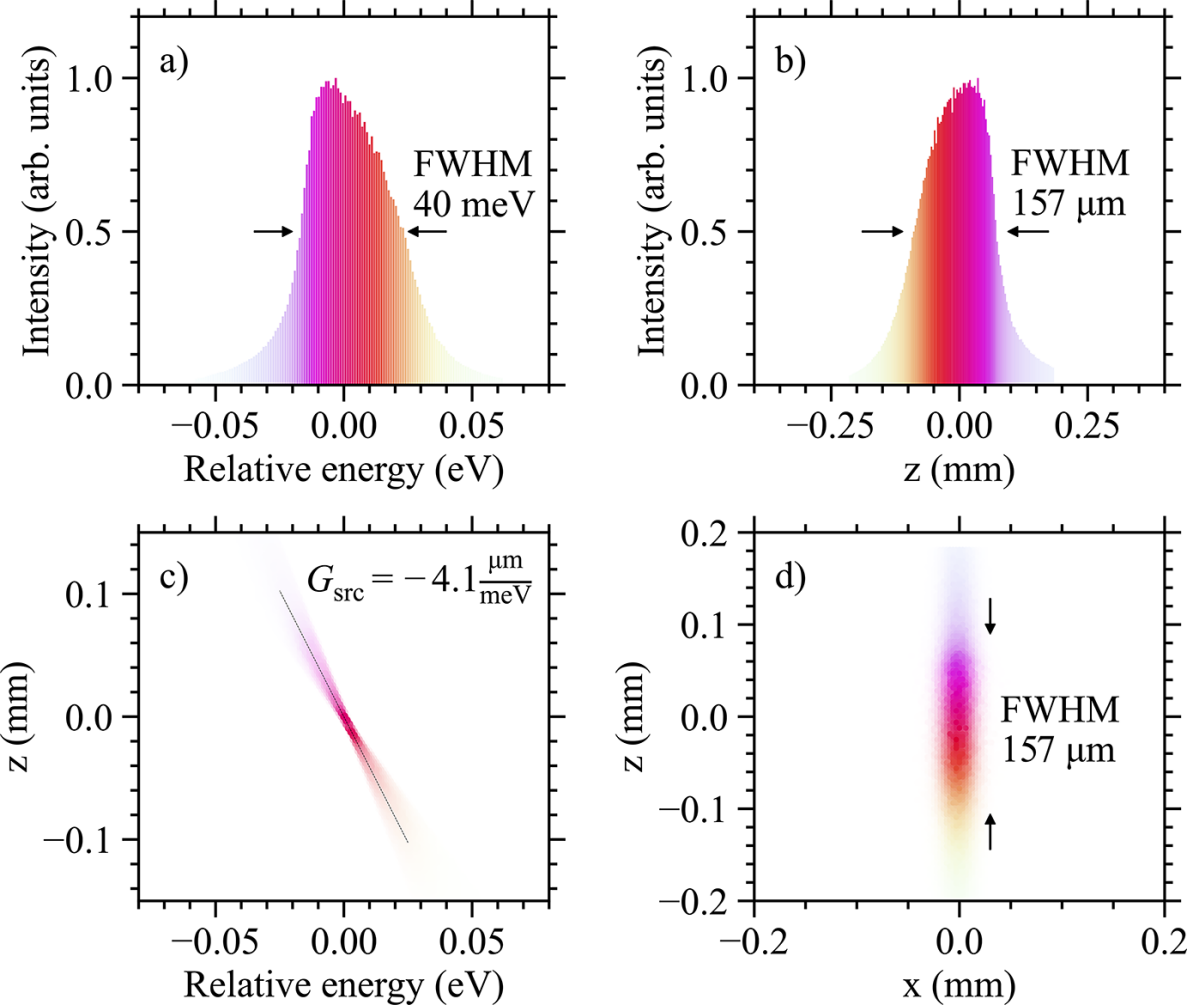
(*a*)–(*d*) Numerical simulations performed for the focused beam using HRM setup 1 at the U *M*_5_-edge. In (*a*) we present the energy profile of the beam with different colors representing different energies. The size of the vertically focused beam is plotted in (*b*), agreeing well with the data in Figs. 5 ▸(*d*) and 6 ▸(*a*). In (*c*) the linear energy dispersion of the source can be seen, with the solid black line representing *G*_src_ = −4.1 µm meV^−1^. Aberrations are observed towards the edges. (*d*) An image of the elongated ‘rainbow’ source. To mimick the horizontally focused beam (*x*-direction) a narrow Gaussian profile (FWHM ∼20 m) was used.

**Figure 10:**
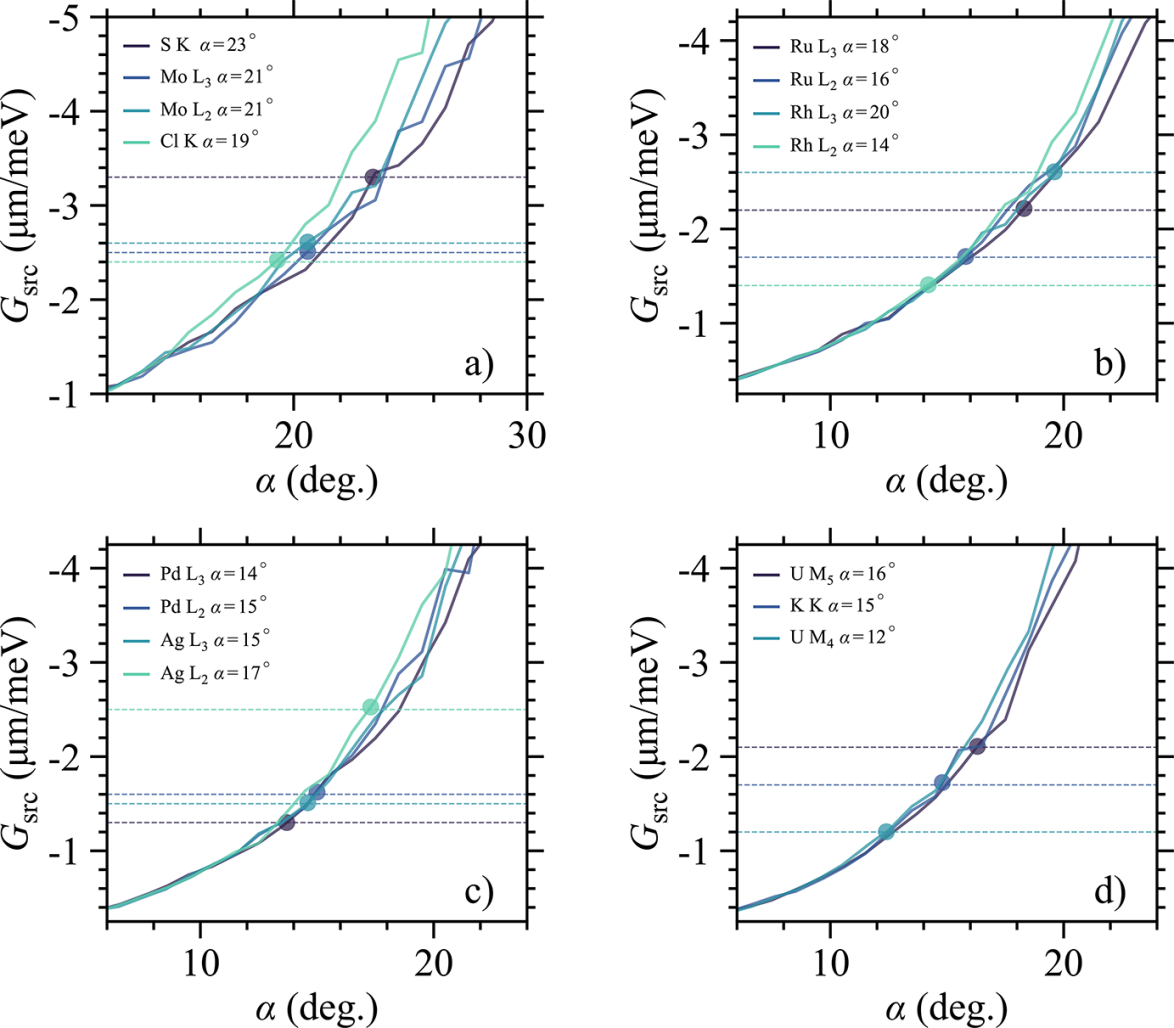
(*a*)–(*d*) Numerical simulations performed for the 15 different edges listed in Table 1 ▸. By tuning α = α_1,2_ = −α_3,4_ of the HRM the linear dispersion rate of the source can be tuned. The dashed horizontal lines present the targeted *G*_src_ values.

**Table 1:** Ray tracing results on the available RIXS edges Analyzers with 30 mm mask (L: LiNbO_3_; S: α-SiO_2_), α = α_1,2_, incident bandwidth Δ*E*_i_, intrinsic spectrometer/analyzer resolution Δ*E*_a_, total energy resolution Δ*E*_tot_, and total energy resolution for a point source 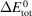.

Edge	*E* (eV)	Analyzer	*G*_det_ (µm meV^−1)^)	α (°)	Δ*E*_i_ (meV)	Δ*E*_a_	Δ*E*_tot_	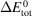
S *K*	2475	L(110)	3.3	24	51	110	110	135
Mo *L*_3_	2520	L(110)	2.5	21	58	130	140	167
Mo *L*_2_	2625	S(110)	2.6	21	56	105	110	122
Cl *K*	2822	S( 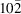 )	2.4	19	58	64	64	91
Ru *L*_3_	2840	S( 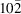 )	2.2	18	63	64	64	97
Ru *L*_2_	2967	S(111)	1.7	16	73	78	78	110
Rh *L*_3_	3005	S(200)	2.6	19	55	77	78	102
Rh *L*_2_	3146	S(200)	1.4	14	81	100	100	134
Pd *L*_3_	3173	S(200)	1.3	13	87	101	105	134
Pd *L*_2_	3330	S( 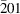 )	1.6	15	73	68	70	105
Ag *L*_3_	3351	S( 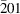 )	1.5	15	77	68	70	108
Ag *L*_2_	3524	S(003)	2.5	17	56	40	40	75
U *M*_5_	3551	S(003)	2.1	16	60	44	44	77
K *K*	3612	S(003)	1.7	15	69	55	55	92
U *M*_4_	3728	S(003)	1.2	13	88	80	80	122

## Data Availability

Data are available upon request.

## Appendix A Simulating the rainbow source

To verify that the approximation for the vertical focus (*l*_src_) in Fig. 5 ▸(*d*) is valid we have also carried out a full ray tracing simulation at the U *M*_5_-edge using the four-bounce inline HRM (setup 1) in combination with a vertical focusing mirror. Results are shown in Fig. 9 ▸. Actual horizontal focusing was not included; instead a narrow Gaussian profile (FWHM ∼20 µm) was used. For a visual aid we have color coded the data following the energy profile in Fig. 5 ▸(*a*). The vertical size of the focused beam is plotted in Fig. 5 ▸(*b*), giving a FWHM of 157 µm in good agreement with Fig. 5 ▸(*d*). In Fig. 5 ▸(*c*) the clear linear energy dispersion can be seen, where the energy of the X-rays gets lower as we move towards the top of the beam. The waist of this diagonal line broadens towards the edges due to abberations discussed by Sánchez del Río & Shvyd’ko (2019 ▸) and Bertinshaw *et al.* (2021 ▸). This takes place because the higher energies have a shorter focal distance while the lower energies have longer—an effect that cannot be avoided when using a focusing mirror.

## Appendix B Expanding to other edges

In Table 1 ▸ we list a few edges that fall within the 2.4–4.0 keV energy range along with possible analyzers (L: LiNbO_3_; S: α-SiO_2_). The analyzers were selected based on performance as well as mechanical limitations of the Rowland spectrometer ( = 78°). The desired linear dispersion rate for each edge is calculated based on equation (1), ranging from the modest value of 1.2 to 3.3 µm meV^−1^. To fulfill equation (2) we tuned the HRM using α = α_1,2_ = −α_3,4_ with the results for all the 15 edges plotted in Fig. 10 ▸. We note that this approach is different from the one in Section 4, where only α_1_ and α_4_ were varied. However, it offers greater tunability and flexibility in optimizing the system. The solid lines are from simulations while the dashed horizontal lines represent the targeted *G*_src_. Numbers from Fig. 10 ▸ are also listed in Table 1 ▸ along with simulated Δ*E*_i_, Δ*E*_a_ and Δ*E*_tot_. In all cases we are able to reach the intrinsic resolution of the spectrometer/analyzer. To facilitate some sort of comparison we also present  for the same incident beam but as a point source (*G*_src_ = 0). The linear dispersion compensation effect is most prominent where Δ*E*_i_ is larger than Δ*E*_a_, such as for the potassium *K*-edge; here the resolution almost doubles if a well focused beam is used. For the edges where the incident bandwidth is significantly smaller, compared with the intrinsic resolution of the spectrometer/analyzer, the dispersion compensation has less of an effect (*e.g.* Mo *L*_2,3_-edges).

For our targeted edges, it is therefore theoretically possible to tune the HRM by selecting crystals with the appropriate asymmetric cuts. However, since α and Δ*E*_i_ are related—increasing the former decreases the latter—it is not possible to freely select the incident bandwidth but we note that by swapping Si(111) crystals in the HRM to Ge(111) one can gain a factor of two in Δ*E*_i_ (twice the flux) while maintaining the desired linear dispersion rate. However, given problems in the past (Bertinshaw *et al.*, 2021 ▸), special care for the crystaline quality of Ge should be taken.
